# Supplementary material for: A Study of the Dielectric Relaxation of Nitrile–Butadiene Rubber, Ethylene–Propylene–Diene Monomer, and Fluoroelastomer Polymers with a Self-Developed Deconvolution Analysis Program
Source: Polymers (Basel). 2025 May 31;17(11):1539. doi: 10.3390/polym17111539 (PMC12157234; doi:10.3390/polym17111539)
Supplement: Supplementary file 1 [file polymers-17-01539-s001.zip › polymers-3566544-supplementary.pdf]

Supplementary Material:

A. Fitting Strategy

In this manuscript, the deconvolution of the obtained dielectric spectra was performed using the self-developed software, "Dispersion Analyzer." The simultaneous fitting of broadband temperature-dependent spectra using multiple combinations of the Havriliak–Negami (HN) equation required careful selection of the optimization algorithm and appropriate constraint settings to ensure iteration stability and convergence.

To address these challenges, "Dispersion Analyzer" provides a variety of configurable fitting options, as illustrated in Figure S1. In Figure S1(b), users can select optimization methods such as Simplex, Hessian-based, or Parallel optimization—as well as define fitting tolerance levels and the maximum number of iterations. The software also supports multi-core parallel processing, where users can specify the number of processor cores to be used for accelerated computation.

Furthermore, users can impose constraints on each fitting parameter within the HN function, and define connectivity constraints between adjacent temperature datasets to ensure physically consistent behavior across temperature. Once the settings are defined, the iterative fitting process is initiated. The quality of the final fit is quantitatively assessed using a Figure of Merit (FOM), which is defined as the sum of squared deviations between the measured and calculated complex permittivity data:

$$FOM = \sum_{1}^{n} [\left(\Delta\epsilon'(\omega_i)\right)^2 + \left(\Delta\epsilon''(\omega_i)\right)^2] \quad \text{(S1)}$$

Where

$\Delta\epsilon'(\omega_i) = \epsilon'_{meas}(\omega_i) - \epsilon'_{fit}(\omega_i)$, $\Delta\epsilon''(\omega_i) = \epsilon''_{meas}(\omega_i) - \epsilon''_{fit}(\omega_i)$

This error metric is implemented flexibly to support different types of discrepancy measures:

Linear: $\Delta y = y_{meas} - y_{fit}$

Logarithmic: $\Delta y = log_{10}(y_{meas} + \delta) - log_{10}(y_{fit} + \delta)$

where δ is an offset added to avoid divergence near zero.

Mixed: combination of linear and logarithmic terms.

The final fit is approved based on one or more of the following criteria:

1. The FOM falls below a user-defined threshold.

2. The optimization algorithm (e.g., Simplex method) converges within a maximum number of iterations.

3. All fitted parameters remain within physically meaningful and predefined bounds.

Error reporting is carried out by explicitly displaying the FOM values in the output tables and graphical interface (the red box in Figure S1(a)). Furthermore, we provide residual plots of real and imaginary components to allow visual inspection of the fitting quality. These combined procedures constitute our quality control framework, ensuring both quantitative accuracy and qualitative reliability of the fit.

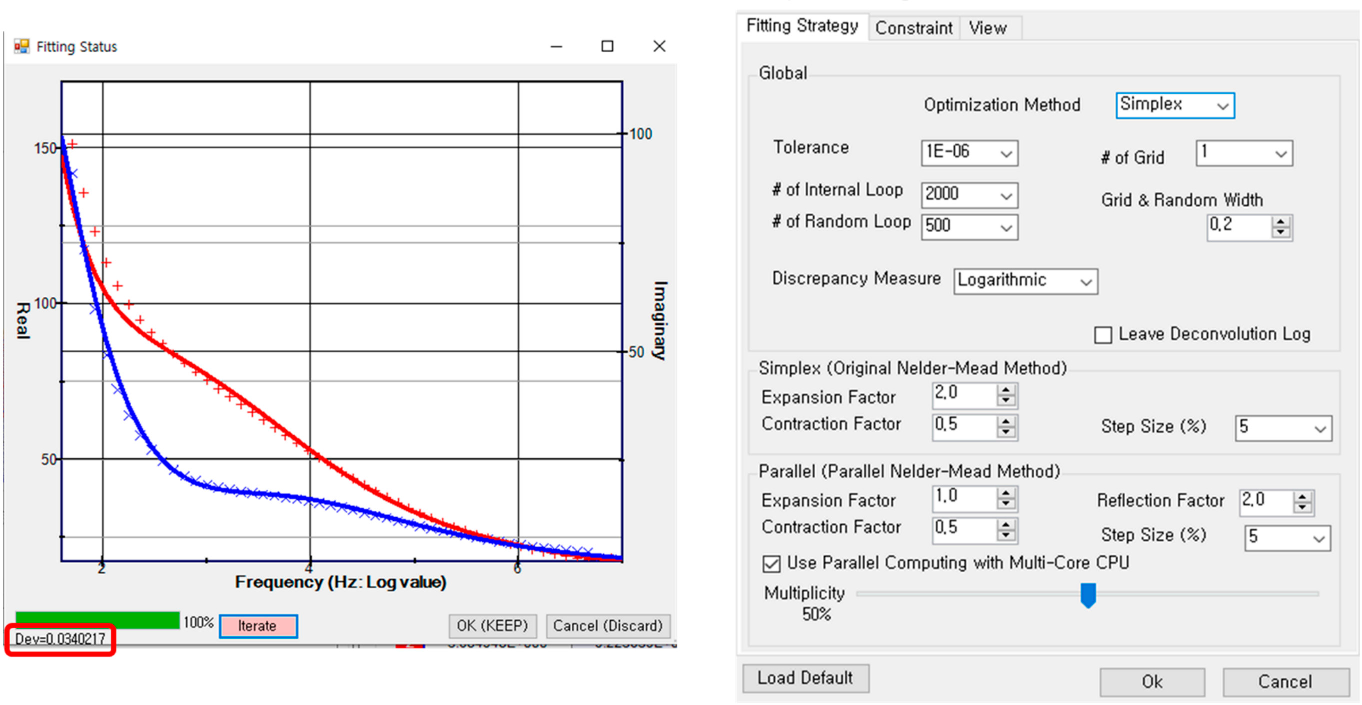

(a) (b)

Figure S1. (a) The screenshot image of fitting status panel. Red cross symbols and solid red lines represent the measured and fitted real parts of the permittivity, respectively, while blue cross symbols and solid blue lines indicate the measured and fitted imaginary parts. The calculated Figure of Merit (FOM), representing the fitting accuracy, is shown in the lower-left corner of the iteration tab. (b) Screenshot of the detailed iteration setup panel. Users can define their fitting strategy by selecting the optimization method (e.g., Simplex, Hessian, or Parallel), the number of iterations, and the number of CPU cores to be used for parallel computation.

Furthermore, the original data were composed of a set of dielectric spectra versus the frequency measured at each temperature. Basically, one isothermal spectrum might be fitted with a single model function such as Eq. (6). However, as seen in the second term in Eq. (6), the main obstacle encountered was the number of relaxation processes that should be introduced. Generally, the imaginary permittivity, ε", which is considered a function of the frequency, has one peak for one relaxation process. The number of relaxation processes can be roughly estimated from the shape of the dielectric spectrum versus frequency. However, many ambiguities exist for determining the number of peaks.

One of these ambiguities emerged because the measurement was basically performed over a restricted range. Introducing peaks was unclear when the center of those peaks were placed on the border or outside of the observable range. Another ambiguity was caused by the presence of errors in the experimental data process, such as instrumental errors. The curve looks like one peak that could also be the composition of two or more overlapping relaxation processes. Thus, it was nearly impossible to deconvolute only one spectrum acquired at a specific temperature without ambiguity. After the deconvolution of each spectrum, unknown parameters such as τ_{HNk} in Eq. (10) were estimated and treated as functions of temperature. For the specific unknown parameter called p, the function $p(\mathrm{T})$ could be made up of a series $\{p_1, p_2, \dots, p_N\}$. The parameter $p(\mathrm{T})$ probably behaves smoothly with respect to temperature variation, except in the restricted narrow region where drastic changes in physical properties occur, similar to the critical temperature. Consequently, we impose an additional penalty ensuring maximization of the smoothness of the series of p, similar to an advanced study by Axelrod et al. [1].

We basically use the least-squares method to find the optimal parameters of a model function, such as Eq. (9). To best fit one dataset of isothermal dielectric spectra, numerical optimization for minimizing the least-squares measurement was performed as follows:

$$S = \sum_i^N \sqrt{\frac{\sum_j^M \left|\varepsilon_i^*(\omega_j) - \varepsilon_{i,j}^*\right|^2}{M}} \qquad \text{(S2)}$$

Here, i is summed to N at each temperature point, j is summed to M at each frequency point, ω_j. $\varepsilon_{i,j}^*$ represents the experimental data, and $\varepsilon_i^*(\omega_j)$ represents the fitting model function. To impose an additional requirement of smoothness on each known parameter, the second-order derivative with respect to temperature was used as an indicator of smoothness. We used the following equation as a penalty function (P):

$$P = \sum_{\{p,q,\dots\}} \sqrt{\frac{\sum_{i=2}^{N-1} |p_{i-1} - 2p_i + p_{i+1}|}{\sum_{i=0}^{N} |p_i|}} \quad \text{(S3)}$$

where the summation inside the root is over all of the temperature points for one kind of parameter and the outside summation is over all of the unknown parameters. The minimization of the objective function, which is the weighted sum of the above two functions, is as follows:

$$F(p, q, \dots) = S + \lambda P \quad \text{(S4)}$$

which was performed via the Nelder–Mead method [2], a commonly used numerical method of nonlinear optimization. In the above equation, λ is an adjustable weighting factor. The Nelder–Mead method adopted here is a heuristic search method that can be used for parameter spaces that have more than a few hundred dimensions. For these, ten or more parameters should be introduced to fit the spectral data of one temperature, and the number of unknown parameters should be 300 or more for all temperatures. For this high-dimensional parameter space, traditional algorithms such as the Hessian matrix method suffer from tremendously long computational times. Figure S2 shows the integrated analysis program and the dialog form for the deconvolution of the spectrum. In Figure S2(a), a screenshot of the main program and the complex permittivity spectrum for each temperature are shown. In Figure 3, the spectrum can be deconvoluted by introducing two HN models with 10 specific parameters, as shown in the table at the bottom righthand side of the panel. In Figure S2(a), all of the parameters are plotted as a function of temperature in the range of −40 °C~100 °C at intervals of 5 °C on the left side of the panel. Several parameters involved in the optimization strategy, such as the weighting factor λ in Eq. (S4), can be adjusted by this dialog window. A flowchart of the developed algorithm is presented in Figure S2(b).

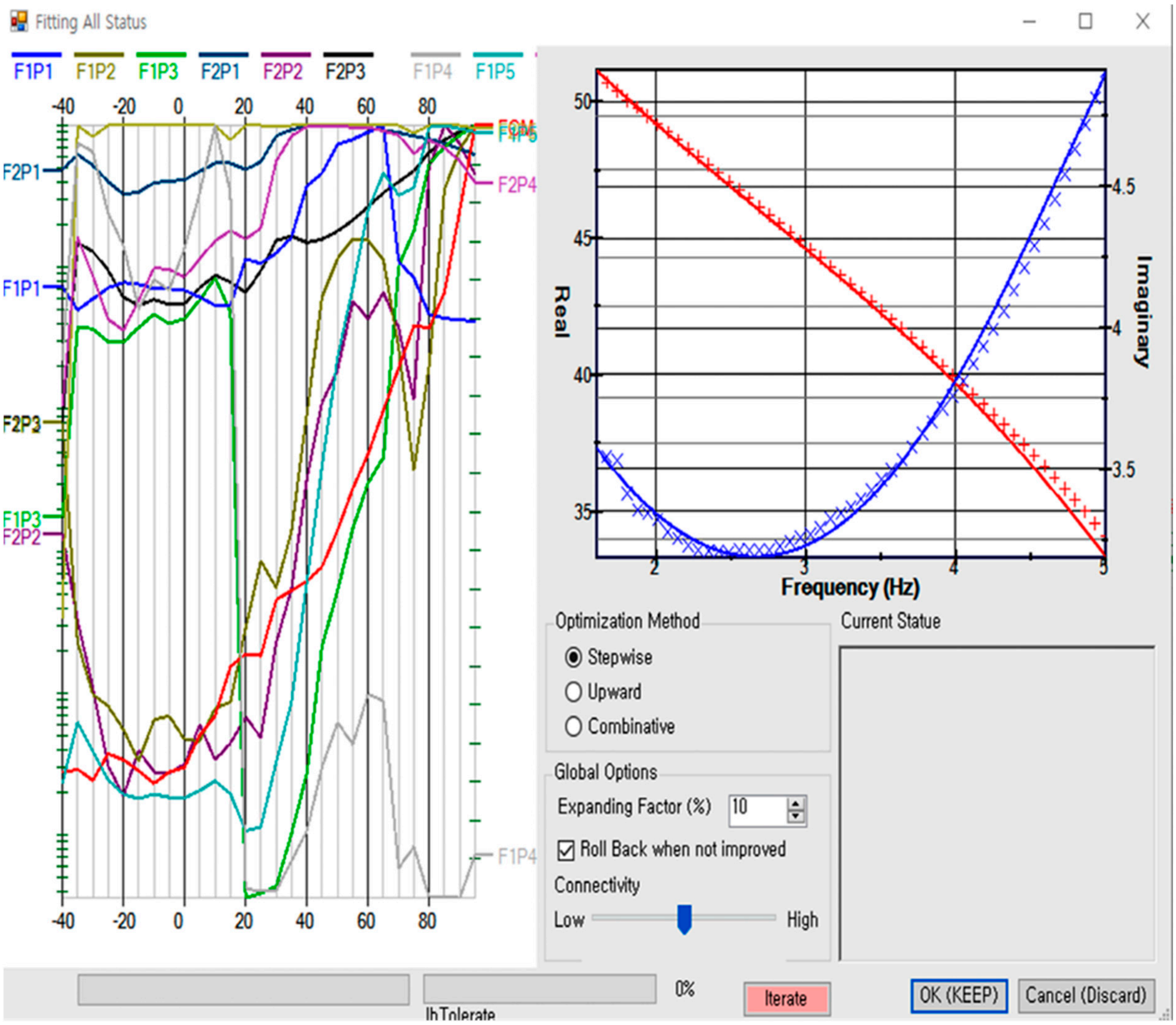

(a)

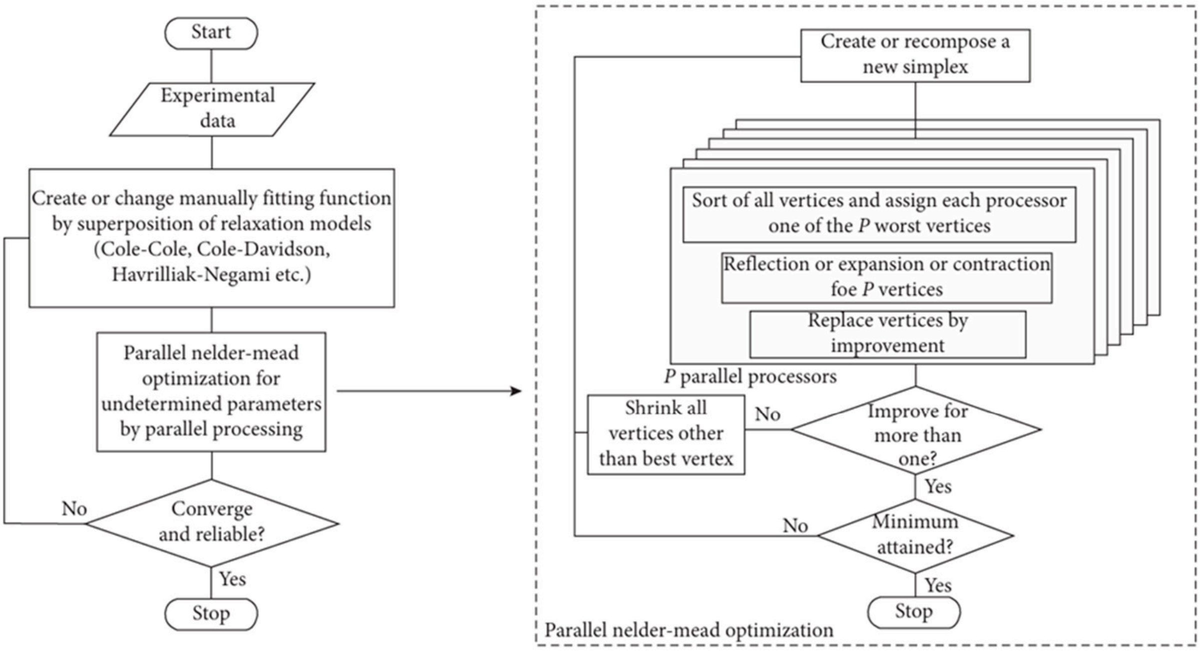

(b)

Figure S2. (a) All of the fitted parameters as imposing smoothness requirements are plotted as a function of temperature; (b) Algorithm of the dispersion analysis program, which employs parallel Nelder–Mead optimization. The entire code of this program is available in the attached supplementary zip file.

B. Complex Dielectric spectra for NBR, EPDM and FKM

The measured complex dielectric spectra for NBR, EPDM, and FKM are displayed in Figure S3(a–f), showing the real and imaginary parts of the permittivity over a broad frequency range at various temperatures. These spectra provide the foundational data for analyzing relaxation processes and evaluating temperature-dependent dielectric behavior in each polymer system.

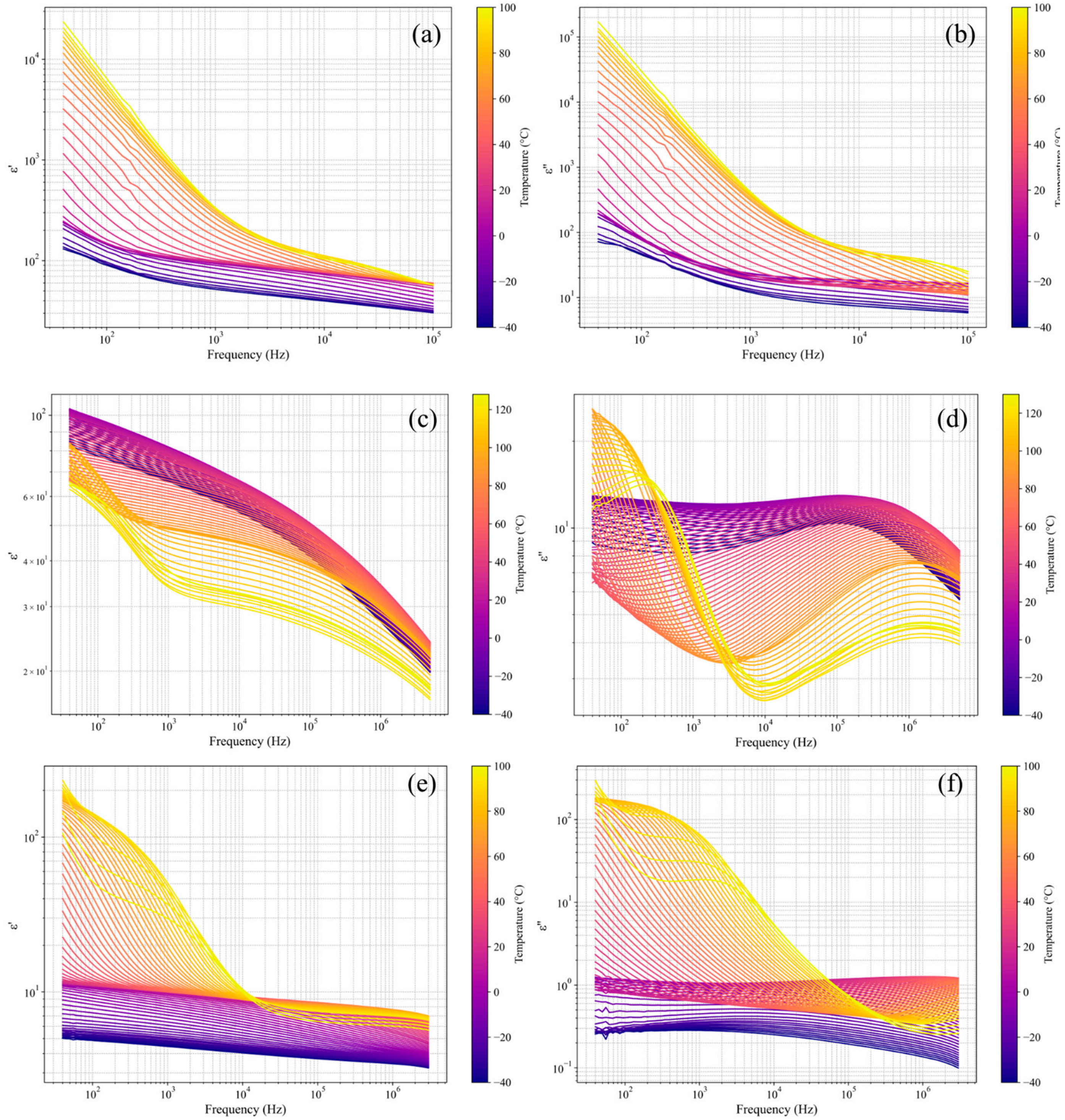

Figure S3. The real and imaginary parts of the complex permittivity for NBR (a, b), EPDM (c, d), and FKM (e, f), respectively.

C. Power law factor N

Figure S4 presents the temperature-dependent behavior of the power-law exponent N, which characterizes the conduction mechanism and electrode polarization effect in the dielectric response. The N parameter was extracted from the global fitting of the complex permittivity spectra using the HN formalism. In Figure S4(a), the N values for the EPDM sample show a gradual increase with temperature, ranging from approximately 0.87 to 0.95 over the investigated temperature range. This trend suggests a moderate enhancement in conduction or electrode polarization effects at

elevated temperatures. In contrast, the FKM sample, shown in Figure S4(b), exhibits relatively stable N values close to unity across most temperatures, with some fluctuation at lower temperatures near 280 K. The near-constant behavior of N implies a less temperature-sensitive conduction process and potentially weaker electrode polarization in the FKM system.

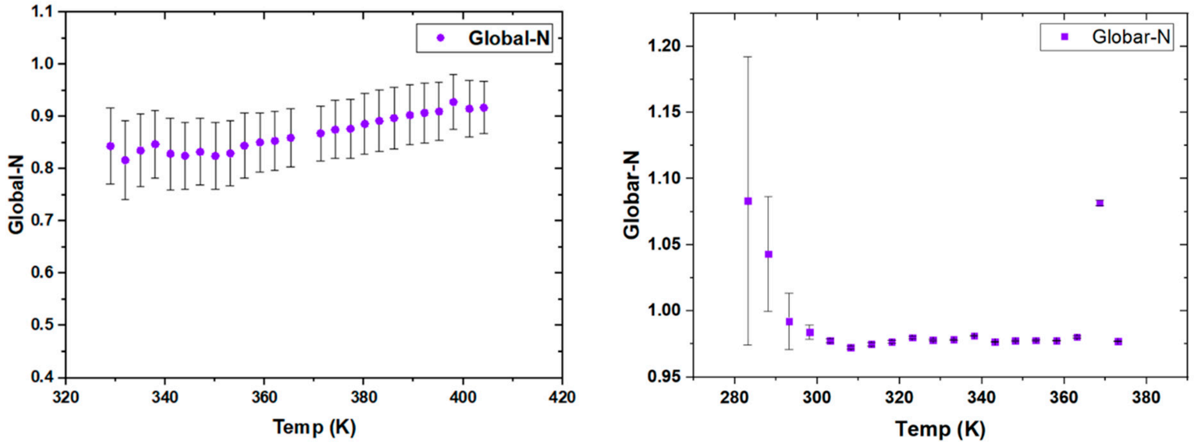

(a) (b)

Figure S4. Temperature dependence of the power-law exponent N for EPDM (a) and FKM (b).

D. References

1. Axelrod, N.; Axelrod, E.; Gutina, A.; Puzenko, A.; Ishai, P.B.; Feldman, Y. Dielectric spectroscopy data treatment: I. Fre-quency domain. Meas. Sci. Technol. 2004, 15, 755–764. https://doi.org/10.1088/0957-0233/15/4/020

2. Nelder, J.A.; Mead, R. A simplex method for function minimization. Comput. J. 1965, 7, 308–313. https://doi.org/10.1093/comjnl/7.4.308
